# Case Report: Pseudo-wide QRS complex tachycardia in an infant with complex congenital heart disease

**DOI:** 10.3389/fped.2025.1643670

**Published:** 2026-01-12

**Authors:** Hualian Li, Jingjing Cong, Tingting Yu, Zhongmin You, Jie Wen, Heng Li

**Affiliations:** 1 Department of Electrocardiography, Maternal and Child Health Hospital of Hubei Province, Wuhan, China; 2 Department of Pediatric Cardiology, Maternal and Child Health Hospital of Hubei Province, Wuhan, China; 3 Department of Pediatric Rehabilitation, Maternal and Child Health Hospital of Hubei Province, Wuhan, China

**Keywords:** congenital heart disease, prolonged PR interval, pseudo-wide QRS tachycardia, R-P fusion, sinus tachycardia

## Abstract

The initial electrocardiogram (ECG) of a 5-month-old infant with complex congenital heart disease (pulmonary valvular stenosis, ventricular septal defects, and patent ductus arteriosus) revealed a regular wide QRS tachycardia at 169 bpm, initially suggestive of ventricular tachycardia (VT) or supraventricular tachycardia (SVT) with aberrant conduction. However, a subsequent ECG obtained during spontaneous heart rate deceleration to 143 bpm showed narrow QRS complexes with discernible sinus P waves and a markedly prolonged PR interval (300 ms). These findings establish the diagnosis of sinus rhythm with biatrial enlargement and first-degree atrioventricular (AV) block. Comparative analysis of these ECGs revealed that the initial tracing did not represent a true wide QRS complex but rather a “pseudo-wide QRS complex” tachycardia. This phenomenon occurs when profound PR prolongation results in concealed sinus P waves overlapping the terminal portion of the preceding QRS complex, thereby mimicking a wide QRS complex. The significant PR interval prolongation, reflecting first-degree AV block, is likely attributable to underlying anatomical abnormalities leading to atrial enlargement and consequent impaired AV nodal conduction. This case report highlights the critical importance of meticulously identifying concealed sinus P waves within wide QRS rhythms to prevent misdiagnosis and inappropriate interventions.

## Introduction

Congenital heart disease (CHD) accounts for nearly one-third of all major congenital malformations, with an estimated global prevalence of 9 per 1,000 live births (1). Common clinical manifestations include patent ductus arteriosus (PDA), atrial septal defect (ASD), ventricular septal defect (VSD), and pulmonary stenosis (PS), which may occur in isolation or in combination. Arrhythmias represent a critical complication in CHD, contributing significantly to morbidity, mortality, and reduced quality of life (1). The electrocardiographic diagnosis of tachycardia in these patients is often challenging. Wide QRS complex tachycardia, typically defined by a QRS duration >120 ms in adults and by age-adjusted norms in children, primarily necessitates differentiation between ventricular tachycardia (VT) and supraventricular tachycardia (SVT) with aberrant conduction (2–4). In this context, we describe a distinct phenomenon termed “pseudo-wide QRS complex tachycardia.” This term refers to a rhythm in which the surface ECG displays an apparently wide QRS complex, not because of delayed ventricular activation (e.g., bundle branch block or VT) but due to superimposition of a prominent P wave—resulting from significant PR interval prolongation—onto the preceding QRS complex. This overlap creates the illusion of QRS widening. This deceptive pattern finds a parallel in adult cardiology, particularly in the setting of myocardial infarction, where P waves hidden within or misinterpreted as part of the QRS complex can lead to misdiagnosis, underscoring a shared electrophysiological principle of wave superposition that can challenge conventional diagnostic algorithms (5, 6).

While this specific “pseudo-wide” mechanism is under-recognized in pediatrics, the broader challenge of misdiagnosing wide QRS tachycardia in patients with CHD is well acknowledged. For instance, in patients who have undergone repair of tetralogy of Fallot, pre-existing bundle branch block can lead to misdiagnosis during tachycardia; however, this scenario represents true QRS widening, in contrast to the apparent widening that is central to our definition. Other mechanisms, such as tremor-induced pseudo-widening in Parkinson's disease, are mechanistically distinct (7). A targeted literature search revealed no prior reports explicitly describing this specific P-wave superposition mechanism in children with CHD. This gap, coupled with the known reduced sensitivity of standard VT-SVT differentiation algorithms in complex CHD, highlights the critical need for heightened awareness. We therefore present the case of a 5-month-old infant with complex CHD (valvular PS, muscular VSD, and ASD) who exhibited “pseudo-wide QRS tachycardia” to elucidate its electrophysiological basis and improve accurate diagnosis.

## Case presentation

A 5-month-old infant with a history of complex congenital heart disease (valvular PS, multifenestrated muscular VSD, and ASD) and a prior hospitalization for pneumonia was admitted with persistent paroxysmal cough, nasal congestion, and rhinorrhea lasting 3 days. The cough was not accompanied by fever, wheezing, or dyspnea. No prior cardiac interventions had been performed.

**Figure 1:**
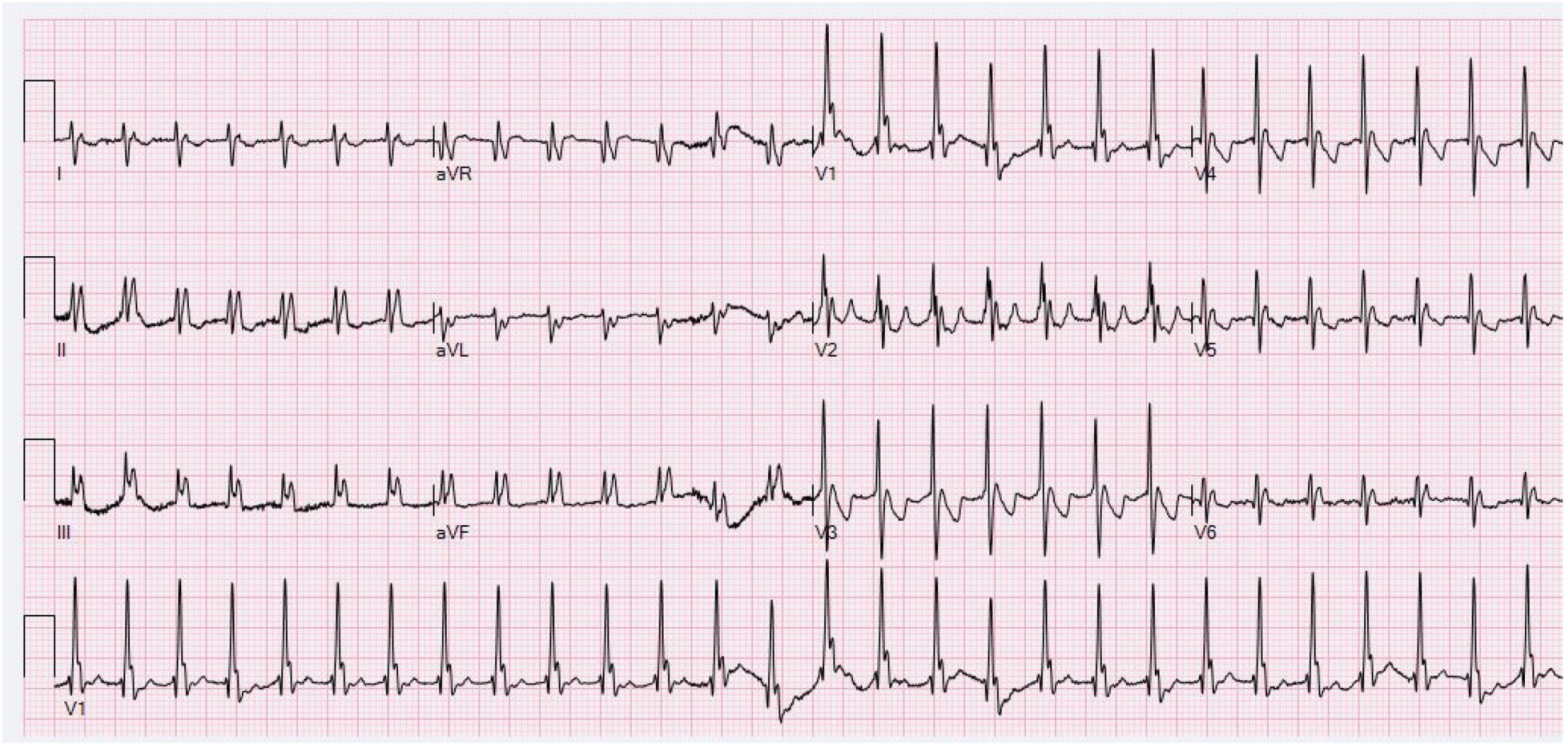
Initial ECG during tachycardia (169 bpm) demonstrating apparent wide QRS complexes with low-amplitude or inverted T waves, suggesting ventricular tachycardia or supraventricular tachycardia with aberrant conduction. P waves are not readily identifiable (paper speed: 25 mm/s, calibration: 10 mm/mV, and filter setting: 100 Hz).

**Figure 2:**
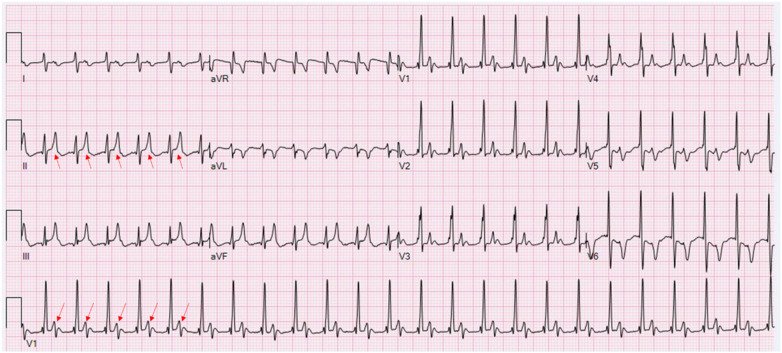
During relative rate deceleration to 143 bpm, a repeated ECG reveals clearly discernible sinus P waves (red arrows), exhibiting positive polarity in inferior leads and negativity in aVR. Key findings include (1) a prolonged PR interval (300 ms), diagnostic of first-degree atrioventricular block; (2) biphasic P waves in V1 (PtfV1 > −0.04 mm·s) and tall P waves in inferior leads (0.5–0.7 mV), consistent with biatrial enlargement; and (3) QRS complexes showing an incomplete right bundle branch block (RBBB) pattern (rsR' in V1; QRS duration < 120 ms) with associated ST depression in leads V5–V6 (paper speed: 25 mm/s, calibration: 10 mm/mV, and filter setting: 100 Hz).

On physical examination, the infant weighed 5.5 kg and had relatively stable vital signs (respiratory rate: 30–40 breaths/min, heart rate: 120–180 bpm, peripheral oxygen saturation: 96%). A grade 3/6 systolic precordial murmur, bilateral coarse crackles, hepatomegaly (1 cm below the costal margin), and poor peripheral circulation of the limbs were noted. Laboratory tests revealed leukocytosis (WBC 17.44 × 10^9^/L, neutrophils 46.7%), elevated CK-MB (40.1 U/L), and markedly elevated NT-proBNP (5,417 pg/mL). The electrocardiographic (ECG) findings are shown in Figures 1, 2. Pathogen screening, arterial blood gas analysis, and electrolyte testing were unremarkable. Chest radiography showed cardiomegaly with features consistent with bronchiolitis.

**Figure 3:**
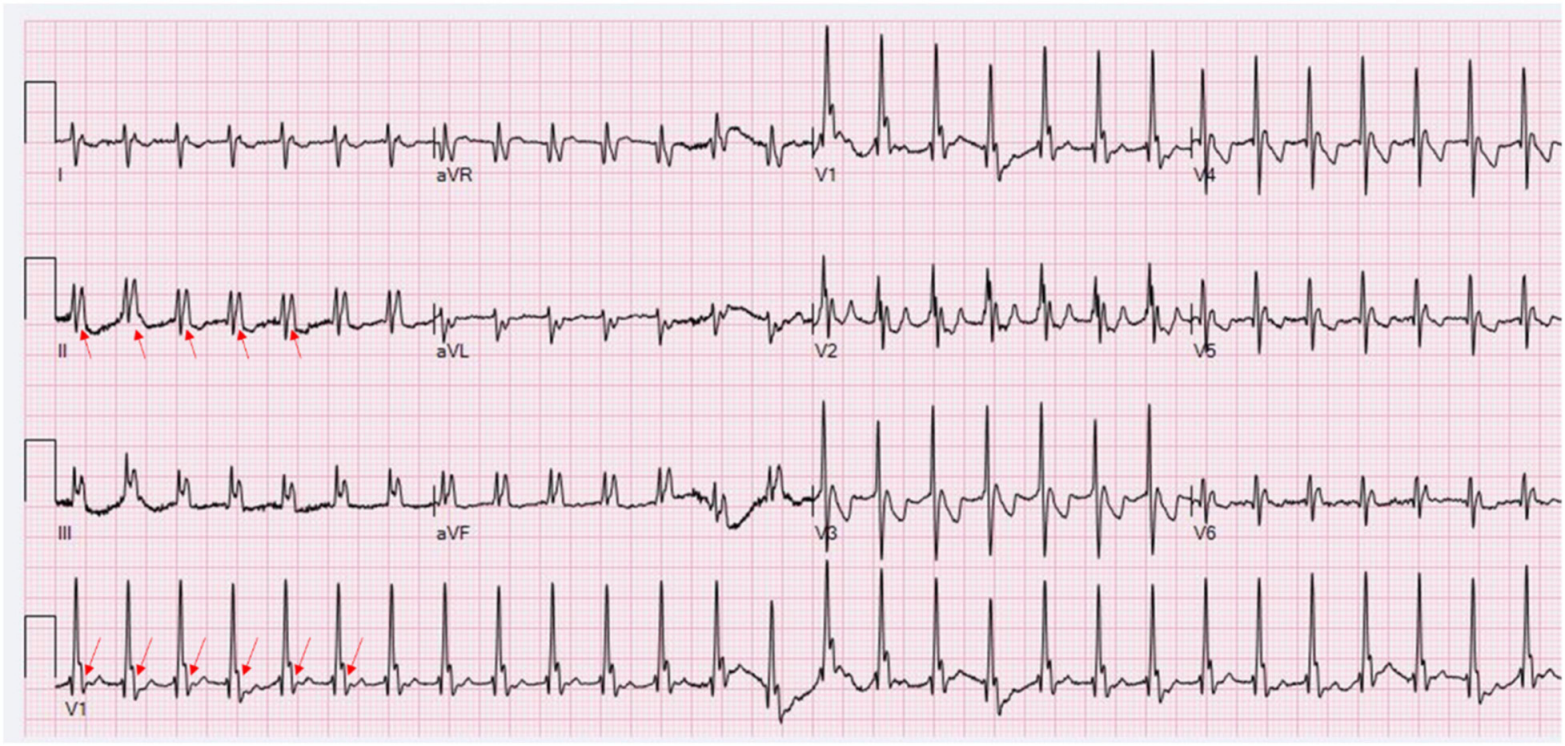
Retrospective analysis of Figure 1 revealing sinus P waves (red arrows) concealed within terminal QRS complexes, most prominently in V1. These P waves exhibit morphological features identical to those in Figure 2, confirming sinus tachycardia at 169 bpm with biatrial enlargement. The significantly prolonged PR interval (300 ms) results in overlap between the sinus P wave and the terminal portion of the preceding QRS complex, thereby generating a pseudo-wide QRS complex—a finding inconsistent with ventricular tachycardia (paper speed: 25 mm/s, calibration: 10 mm/mV, and filter setting: 100 Hz).

Upon admission, the infant's respiratory symptoms in the context of known heart disease raised immediate concern for acute cardiac decompensation rather than a simple respiratory infection. Given the clinical signs of fluid overload, fluid restriction was initiated immediately, targeting a maintenance volume of approximately two-thirds of the standard requirement for weight. Empirical antibiotic (cefotiam) therapy was initiated due to current leukocytosis, considering the potential for a bacterial infection triggering decompensation. Definitive heart failure therapy was deferred until confirmatory data were obtained, after which intravenous furosemide (1 mg/kg/day) was administered for diuresis and oral digoxin (5 μg/kg/day) was initiated for inotropic support. Given the sinus origin of the tachycardia, antiarrhythmic therapy was deemed unnecessary. The patient was closely monitored for vital signs, perfusion, and fluid balance via strict intake/output tracking. Serial ECGs and laboratory tests, including electrolytes, renal function, and NT-proBNP, were also performed.

The patient's condition improved over the following 48–72 h, with resolution of pulmonary crackles and reduction in hepatomegaly. However, the family elected to transfer the patient to another facility, resulting in discharge against medical advice before completion of the planned therapeutic course. A structured follow-up plan for ongoing heart failure management and surgical evaluation was provided but ultimately not pursued, precluding assessment of long-term outcomes.

## Discussion

**Figure 4:**
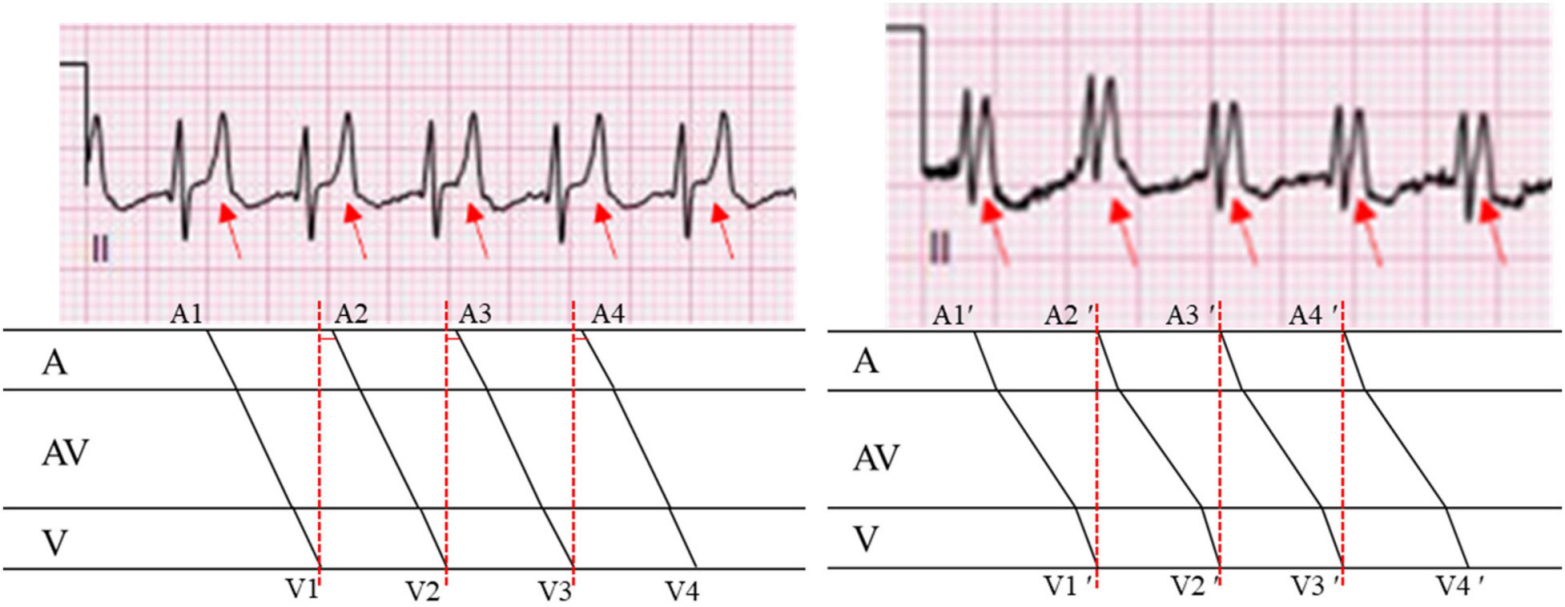
Comparison of the left and right ladder diagrams revealing a shortened interval between V1 and A2 (marked by a solid red line), which is attributable to a markedly prolonged PR interval. In contrast, no interval is present between V1' and A2', indicating that the atrial activity (P wave) overlaps with the preceding ventricular activity (QRS complex).

Our case presents a distinctive diagnostic challenge involving an infant with complex CHD who exhibited a wide-complex tachycardia on the initial ECG (Figure 1). The subsequent tracing (Figure 2) proved pivotal, revealing that the perceived “wide QRS” was, in fact, an illusion. The emergence of narrow QRS complexes with clearly discernible P waves at a slightly slower rate, together with the absence of AV dissociation or fusion beats, definitively excluded VT. Comprehensive analysis of P-wave morphology in Figure 2—positive in the inferior leads, negative in aVR, and biphasic (positive–negative) in V1—was inconsistent with atrial tachycardia, junctional tachycardia, or typical short RP (RP < PR) tachycardias, such as slow–fast atrioventricular nodal reentrant tachycardia (AVNRT) or orthodromic atrioventricular reentrant tachycardia (AVRT). In typical slow–fast AVNRT, P waves are usually buried within or at the terminal portion of the QRS complex, presenting as pseudo-S waves in inferior leads or a pseudo-R' in V1. In contrast, in orthodromic AVRT, retrograde P waves typically occur early in the ST segment (2, 8). Therefore, the rhythm in Figure 2 is best diagnosed as sinus tachycardia with a markedly prolonged PR interval and biatrial enlargement. This interpretation clarifies the initial rhythm in Figure 1. Although the P waves in Figure 1 are obscured, their timing and implied morphology align with those observed in Figure 2. Thus, Figure 1 primarily represents sinus tachycardia with first-degree AV block and biatrial enlargement. The “pseudo-wide QRS complex” in Figure 3 resulted from superimposition of a sinus P wave onto the terminal portion of the preceding QRS, a direct consequence of extreme PR prolongation. Specifically, while sinus impulses occur at the expected rate, PR prolongation significantly delays ventricular activation such that the subsequent QRS complex is shifted later in time, allowing the next sinus P wave to overlap the terminal portion of the preceding QRS complex (as shown in Figure 4).

The profound PR prolongation observed in this infant stems synergistically from his specific CHD anatomy. First, the presence of a significant muscular VSD and ASD creates a volume-overload state. This chronic volume loading, particularly of the atria due to left-to-right shunting, leads to biatrial enlargement. From an electrophysiological perspective, atrial enlargement stretches and compromises interatrial and intra-atrial conduction pathways, thereby prolonging the P-wave duration and increasing the time required for the sinus impulse to reach the atrioventricular node (AVN). Second, valvular PS imposes a chronic pressure overload on the right ventricle, which is transmitted back to the right atrium, further contributing to right atrial enlargement and hypertrophy. An enlarged and hypertrophied atrium can physically distort the anatomical environment of the AVN and increase the distance that the cardiac impulse must travel. In addition, the AVN itself may be directly affected in CHD. Chronic hemodynamic stress, together with possible direct architectural influences from adjacent chamber enlargement, can promote fibrosis and alter the electrophysiological properties of the AVN, intrinsically slowing conduction. Thus, the marked PR prolongation in this case likely reflects a composite mechanism: delayed intra-atrial conduction due to biatrial enlargement combined with impaired AV nodal conduction, both stemming from the underlying congenital defects.

While the structural abnormalities provide the most plausible explanation for the conduction delay, we systematically considered alternative etiologies. Post-surgical scarring was ruled out, as the patient had not undergone any cardiac procedures before the ECG recording. Common inflammatory or infectious etiologies were deemed unlikely given the absence of supportive symptoms or laboratory findings. In addition, the patient was not receiving any medications known to affect AV conduction. Although connective tissue disorders, channelopathies, or an undiagnosed syndrome cannot be entirely excluded, the lack of a relevant family history and extracardiac manifestations makes these possibilities less likely. Degenerative conduction system disease is exceedingly rare in infants. Furthermore, the stability of the PR interval on serial ECGs, without progression to higher-grade AV block, supports a non-progressive, hemodynamically mediated etiology rather than a progressive intrinsic pathology.

Accurate identification of this “pseudo-wide QRS” pattern carries critical therapeutic implications. Misinterpretion of this rhythm as VT could lead to inappropriate and potentially harmful interventions. Administration of antiarrhythmic drugs, which possess adverse inotropic and pro-arrhythmic effects, could precipitate hemodynamic collapse in an infant already compromised by complex CHD. Unnecessary electrical cardioversion would expose the child to the risks associated with sedation and anesthesia, as well as the trauma of the shock itself, all for a rhythm that is fundamentally sinus tachycardia and requires no such intervention. Furthermore, misdiagnosis of VT might lead to inappropriate referrals for invasive electrophysiological studies or even consideration of implantation of a cardioverter-defibrillator in this infant. This case highlights how even a mild respiratory infection can rapidly precipitate heart failure in infants with underlying cardiac disease, underscoring the importance of early recognition, prompt diagnosis, and timely targeted therapy. However, the patient's self-discharge highlights important challenges related to medical adherence and family communication, which may limit the generalizability of case management.

## Conclusion

This case report describes a phenomenon termed “pseudo-wide QRS complex tachycardia” in an infant with complex CHD, driven by extreme PR prolongation secondary to biatrial enlargement and AV nodal conduction delay. This phenomenon must be distinguished from true wide-complex tachycardias, as management strategies differ fundamentally. A high index of suspicion, meticulous analysis of P-wave morphology across sequential tracings, and an understanding of the unique electrophysiological substrates associated with CHD are essential for accurate diagnosis. Future studies utilizing invasive electrophysiological assessment in similar patients may help localize the site of conduction delay more precisely, and genetic analyses could uncover potential syndromic associations.

## Data Availability

The original contributions presented in the study are included in the article/Supplementary Material; further inquiries can be directed to the corresponding author.
